# The Role of Interleukin-18 After Spinal Cord Injury: Mechanisms and Therapeutic Potential

**DOI:** 10.3390/cells15111011

**Published:** 2026-05-31

**Authors:** Luke J. Bolstad, Mia J. LaRico, Thomas S. Zanovich, Grant R. Keith, Amgad S. Hanna, Daniel J. Hellenbrand

**Affiliations:** 1 Department of Neurosurgery, School of Medicine and Public Health, University of Wisconsin-Madison, Madison, WI 53706, USA; ljbolstad@wisc.edu (L.J.B.); larico@wisc.edu (M.J.L.); thomaszan722@gmail.com (T.S.Z.); gkeithmd@gmail.com (G.R.K.); 2 Department of Biomedical Engineering, University of Wisconsin-Madison, Madison, WI 53706, USA

**Keywords:** spinal cord injury (SCI), Interleukin-18 (IL-18), microglia-astrocyte crosstalk, NLRP3 inflammasome, neuroinflammation

## Abstract

Spinal cord injury (SCI) triggers a secondary injury cascade characterized by neuroinflammation, reactive gliosis, and neuronal apoptosis. While many pro-inflammatory cytokines contributing to this cascade reach peak upregulation within 24 h, Interleukin-18 (IL-18) exhibits a delayed upregulation profile, typically peaking 7 days post-injury. This review examines the temporal regulation and cell-specific roles contributing to the rise in IL-18 after SCI. Following primary insult, damage-associated molecular patterns prime and activate the NLRP3 inflammasome, which in turn drives latent IL-18 secretion. Cellularly, microglia function as the primary producers of IL-18 via the TLR4/p38-MAPK pathway, while astrocytes serve as the primary responders through IL-18R/p65-NF-κβ signaling. The microglia-astrocyte cross-talk propagates reactive gliosis, drives neuropathic pain, facilitates neuronal loss, and potentially contributes to the formation of the astrocytic border. Targeted therapeutic interventions such as upstream inhibition of NLRP3 inflammasome assembly or direct IL-18 neutralization successfully mitigate neuroinflammation. By either inhibiting NLRP3 inflammasome activation or directly neutralizing IL-18, these treatments shift the microglial toward a protective state, restrict histological damage, and significantly improve functional recovery.

## 1. Introduction

Spinal cord injury (SCI) remains a global health crisis, with approximately 14.5 million individuals currently living with the condition and the prevalence expected to surpass this number by 2050. Specifically, the prevalence within North America in 2021 was roughly 15 persons per 100,000 people per year [1]. After the primary insult, the injury environment, characterized by the release of inhibitory molecules, the formation of an astrocytic border, and a lack of intrinsic regenerative capacity, strips individuals of motor, sensory, and autonomic functions below the level of injury [2]. The loss of function leads to many secondary complications such as chronic pain (57%), pneumonia, urinary tract infections, spasticity, and the development of pressure sores [3,4]. Beyond physical tolls, these deficits place psychosocial burdens on the affected individuals and their families, manifesting as depression, anxiety, and decreased quality of life [5,6].

Spinal decompression and stabilization within the first 24 h of injury is a common practice. The presence of comorbidities, poly-trauma, and baseline severity of the injury play a significant role in postoperative outcomes. Limited access to specialized spinal surgeons often delays decompression, exacerbating tissue damage and worsening long-term prognoses [7]. Historical attempts at broad immunosuppression, such as high-dose methylprednisolone sodium succinate, failed to demonstrate consistent clinical efficacy, highlighting the challenges of translating preclinical therapeutics into clinical therapeutics [8]. The shortcomings of current therapeutic strategies and the increasing prevalence of SCI underscore the necessity of moving toward highly specific, targeted molecular interventions that address bottlenecks in the secondary injury cascade [1,8].

Several cell types are involved in the secondary injury cascade following SCI: neutrophils, blood-borne macrophages, T-lymphocytes, microglia, and astrocytes [9]. During the acute phase of injury, resident microglia, astrocytes, and peripheral neutrophils are recruited to the injury site [9,10]. Microglia then switch from a protective to a proinflammatory state in response to damage-associated molecular patterns (DAMPs). Proinflammatory microglia secrete cytokines such as Tumor Necrosis Factor-α (TNF-α), Interleukin-1β (IL-1β), and Interleukin-6 (IL-6), further recruiting immune cells to the injury site [11,12,13]. The aforementioned cytokines all reach peak upregulation within 24 h after injury, creating a harsh inflammatory environment [12]. This environment further propagates the cascade, increasing proliferation of microglia and astrocytes, while continuing to recruit immune cells from the periphery [11,12]. Throughout the cytokine storm, infiltrating neutrophils release reactive oxygen species (ROS), reactive nitrogen species, and other enzymes, which cue surrounding cells to undergo apoptosis [14,15,16].

Preclinical animal studies have demonstrated that anti-inflammatory cytokines can reduce inflammation, minimize secondary tissue damage, and significantly improve hindlimb function [17,18,19,20,21,22]. Collectively, these findings underscore the detrimental effects of secondary injury and highlight the therapeutic potential of strategies aimed at attenuating post-SCI inflammation.

While most proinflammatory cytokines reach their peak upregulation within the first 24 h after injury, our group observed that Interleukin-18 (IL-18), a pro-inflammatory cytokine belonging to the IL-1 superfamily, reached peak upregulation 7 days post-injury (DPI) [12,23]. Unlike its pro-inflammatory counterparts that peak acutely, IL-18 exhibits a delayed upregulation timeline, necessitating a closer examination of its unique structural processing and activation pathways. To understand its inflammatory profile, this review examines IL-18′s involvement in inflammation after traumatic SCI. Specifically, this review focuses on delineating cells involved in its upregulation, the temporal regulation of IL-18, and evaluating therapeutic strategies that have mitigated secondary damage and suppressed the endogenous production of IL-18.

## 2. IL-18 Overview

Initially discovered in 1989, IL-18 was first known as “Interferon-γ-inducing factor” or “IGIF” due to its ability to amplify Interferon-γ (IFN-γ) production in T helper cells (Th1) [24,25]. IL-18 belongs to the IL-1 family and shares similar activation mechanisms to IL-1β, as the two are synthesized as biologically inactive precursor proteins and are processed by the same cysteine protease, caspase-1 [26,27]. The inactive precursor, pro-IL-18, has a molecular weight of 24 kDa and remains localized to the cytoplasm until it is cleaved into its active form. Once cleaved, the 18 kDa active form of IL-18 is secreted into the extracellular space [27]. This cleavage process of IL-18 is tightly regulated by the assembly of protein complexes known as inflammasomes.

### 2.1. Inflammasome Processing

The inflammasome pathway is critical in maturation IL-18 as it mediates caspase-1 activation. Although many inflammasomes can activate caspase-1, the NOD-like receptor family pyrin domain containing 1 (NLRP1) inflammasome and the NOD-like receptor family pyrin domain containing 3 (NLRP3) inflammasome are the two most involved in central nervous system (CNS) trauma, like traumatic brain injury (TBI) or SCI [28].

The NLRP1 inflammasome is rapidly upregulated after CNS trauma due to the inflammasome existing as a pre-assembled complex in motor neurons [29,30]. Under healthy conditions, the complex comprises NLRP1, apoptosis-associated speck-like protein containing a caspase recruitment domain (ASC), and X-linked inhibitor of apoptosis protein (XIAP). Upon injury, XIAP is rapidly cleaved, and its inhibitory control is abolished as early as 6 h after injury, allowing for recruitment of caspase-1 to the complex for early maturation of IL-1β and IL-18 [29]. In rats who underwent T-12 SCI, NLRP1, caspase-1, and ACS proteins significantly increase 18 to 24 h after injury, coinciding with the early rise in IL-1β post-SCI [30].

**Figure 1:**
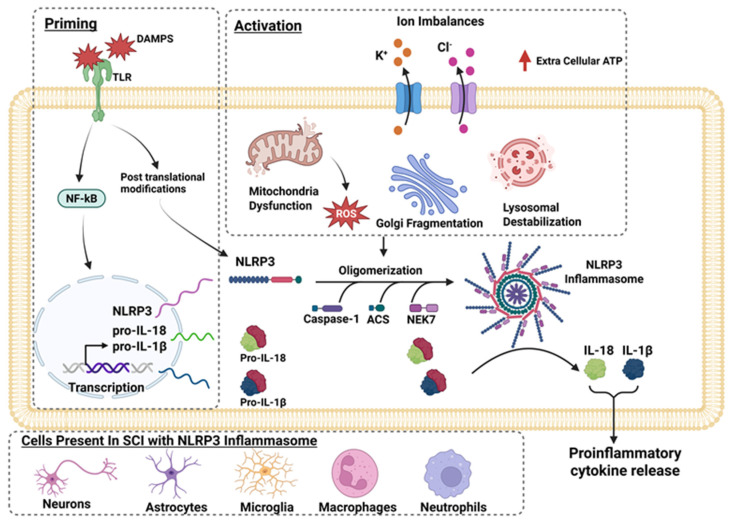
Activation and priming of NLRP3 inflammasome. The NLRP3 inflammasome processes IL-18 following priming and activation. Priming is induced by DAMPS binding to TLRs. Upon binding, the NF-κβ pathway is activated, inducing the transcription of pro-IL-18, pro-IL-1β, NLRP3 mRNA, as well as post-translational modifications to the NLRP3 protein. Following priming, activation occurs through the disruption of basal ion concentrations, causing lysosomal destabilization, Golgi fragmentation, and mitochondria dysfunction, with the latter resulting in the production of ROS. These factors induce the assembly of the NLRP3 inflammasome and subsequent conversion of pro-IL-18 and pro-IL-1β into their mature forms [36,37]. Once mature, IL-18 and IL-1β are secreted into the extracellular matrix [27]. The NLRP3 inflammasome is produced by neurons, astrocytes, microglia, macrophages, and neutrophils [32,33,34]. Figure created with BioRender 2026.

Unlike NLRP1, the NLRP3 inflammasome is not preassembled and is only marginally detectable within the uninjured spinal cord [31]. However, once CNS trauma occurs, the NLRP3 inflammasome is found within neutrophils, macrophages, neurons, microglia, and astrocytes, displaying peak upregulation at later time points compared to NLRP1, 7 DPI [32,33,34]. Notably, the peak upregulation of the NLRP3 protein coincides with our finding of peak upregulation of IL-18 post-SCI and Yatsiv’s findings post-TBI [12,35]. Assembly of the NLRP3 inflammasome is induced by two steps: priming and activation (Figure 1). Priming is triggered by DAMPs present after CNS trauma. These DAMPs bind to toll-like receptors (TLRs) on the surface of cells in the SCI milieu, which upregulate the transcription of NLRP3 and pro-IL-18 through the Nuclear Factor-κβ (NF-κβ) signaling pathway [36]. Following priming, the NLRP3 protein undergoes post-translational modification, and the inflammasome pathway is activated by the disruption of basal ion concentration gradients and increased extracellular ATP, which occurs after trauma [36,37]. These cues further cause the formation of intracellular ROS through lysosomal destabilization, Golgi fragmentation, and mitochondria dysfunction. Once primed and activated, NLRP3 proteins oligomerize and recruit ASC, which mediates the conversion of pro-caspase-1 into its catalytically active form, caspase-1 [36,37]. The formation of this inflammasome further recruits NIMA-related kinase 7 (NEK7), which mediates interactions between adjacent NLRP3 subunits [37]. Assembly of this complex enables mature caspase-1 to convert pro-IL-18 into its active form, allowing the inflammatory cytokine to be secreted out into the extracellular matrix (ECM). Once secreted, IL-18 does not require a carrier protein as it is naturally soluble in the ECM [38]. In the ECM, IL-18 can interact with two possible binding partners, the IL-18 binding protein (IL-18BP) or directly bind to its receptor (IL-18R).

### 2.2. Interleukin-18 Binding Proteins

**Figure 2:**
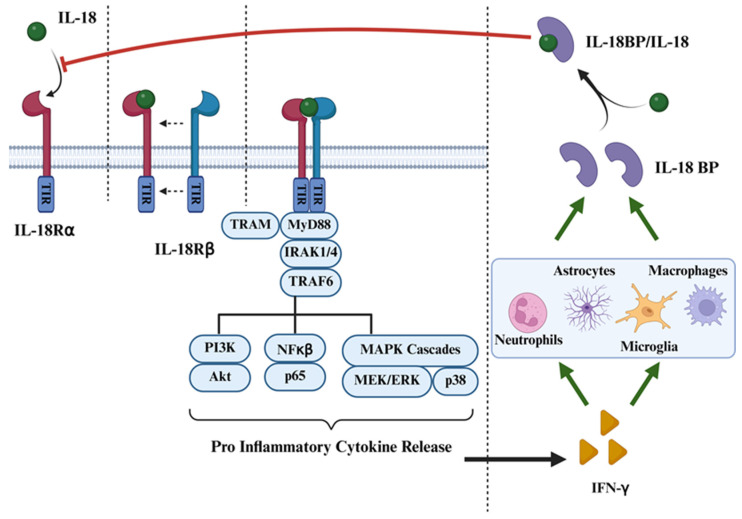
IL-18 binding, activation, and regulation. Free IL-18 initially binds to IL-18Rα. Once bound, IL-18Rβ is recruited, forming a high-affinity complex and juxtaposing intracellular TIR domains, initiating downstream signaling [41,47,48,49]. Activation of PI3K, NF-κβ, and MAPK pathways leads to the release of pro-inflammatory cytokines, including IFN-γ [41,48,50]. IFN-γ production, in turn, acts as a negative feedback loop as IFN-γ stimulates the production of IL-18BP via neutrophils, astrocytes, microglia, and macrophages [42,43,44,45]. IL-18BP binds IL-18 with greater affinity than IL-18R, inhibiting the downstream effects of IL-18R activation [40,41]. Figure created with BioRender 2026.

The IL-18BP is a soluble group of proteins that act as “decoy” receptors, which are encoded on a separate gene from the IL-18R [39]. The binding proteins’ primary function is to bind and neutralize free IL-18, preventing the cytokine from binding to its receptor and triggering an inflammatory response (Figure 2). The binding proteins rely on their high binding affinity to IL-18 to execute their regulatory function. Binding kinetic studies of human IL-18 to human IL-18BP have yielded low dissociation between the two (Kd = 0.4 nM) [40]. On the contrary, the dissociation between human IL-18 and the IL-18Rα chain has higher values (Kd = 69 nM) [41]. Neutrophils, macrophages, microglia, and astrocytes have all been shown to produce IL-18BP [42,43,44,45]. The production of the protein is stimulated by upregulation of IFN-γ [43]. While IL-18BP production is induced by IFN-γ, IL-18BP acts as a negative feedback regulator on the production of IFN-γ, as the binding protein has been shown to abolish IL-18 induction of the NF-κβ pathway and production of IFN-γ in vitro [46]. Notably, miR-92b-5b is implicated in the upregulation of IL-18 BP after SCI, as suppression of this miR after SCI has been shown to increase IL-18BP, corresponding with decreases in IL-18 [45]. When IL-18BP levels fall, free IL-18 is able to bind to its receptor.

### 2.3. IL-18 Receptor and Signaling Pathway

IL-18 differs from the other IL-1 proinflammatory cytokines by its use of specialized transmembrane receptors, IL-18Rα and IL-18Rβ, rather than utilizing a shared accessory protein like the rest of the IL-1 family [41]. However, their systems also consist of similar components wherein they both have a ligand-binding component and a signaling component. In the IL-18R complex, the IL-18Rα chain is the binding subunit and the IL-18Rβ chain is the signaling unit (Figure 2) [51,52,53]. IL-18 contains three active sites; sites I and II interact with IL-18Rα, and site III interacts with IL-18Rβ [47,54]. Once the binding process is initiated, IL-18 is first recognized by IL-18Rα at the two binding sites through surface area interactions facilitated by N-linked glycans, forming a low-affinity complex. IL-18Rβ is then recruited to form a stable heterotrimeric complex, creating a high-affinity binding state [41,47]. It is not until this heterotrimeric complex is formed that the signal transduction pathway commences.

Upon ternary complex formation between IL-18, IL-18Rα, and IL-18Rβ, the Toll/IL-1 receptor (TIR) domains on the cytoplasmic side of each receptor chain are juxtaposed, and the signal transduction pathway begins (Figure 2). Once TIRs are aligned, they recruit myeloid differentiation primary response protein 88 (MyD88) with the help of its sorting adaptor, TRIF-related adaptor molecule (TRAM) [48,49]. MyD88 further recruits IL-1R-associated kinase 1(IRAK1) and IL-1R-associated kinase 4 (IRAK4) to the complex, subsequently interacting with Tumor Necrosis Factor Receptor-Associated Factor 6 (TRAF6), enabling the activation of Phosphoinositide 3-kinase (PI3K), NF-κβ, and Mitogen-Activated Protein Kinase (MAPK) pathways [41,48,50]. In the context of SCI, the activation of these pathways induces the production of many proinflammation cytokines such as IFN-γ. Cell-specific activation and downstream effects will be discussed in the following section.

## 3. Cell-Specific Roles in IL-18 Signaling and Modulation

Many cell types within the immune system and CNS uniquely contribute to the inflammatory cascade by regulating IL-18 levels during the secondary injury phase. Identifying specific cellular sources and their subsequent interactions remains essential for decoding the mechanisms of IL-18-mediated inflammation after SCI.

### 3.1. Neutrophils

Among the peripheral immune cells infiltrating the spinal cord parenchyma, neutrophils are the most abundant early responders and reach peak concentration at 24 h after injury [14]. These cells possess the NLRP3 inflammasome and constitutively produce both pro-IL-18 and mature IL-18 [33,55]. Recent research mapping the cellular sources of IL-18BP in healthy mice identifies circulating neutrophils as a primary source of the binding protein, suggesting these cells play a critical role in regulating the IL-18/IFN-γ axis [42]. Furthermore, neutrophils also express both IL-18Rα and IL-18Rβ chains [56]. Upon binding to its receptor complex, IL-18 triggers the p38-MAPK, MEK/ERK, p65-NF-κβ, and PI3K/Akt signaling pathways [57]. The activation of these pathways initiates a broad spectrum of immune responses by neutrophils, including the priming and enhancement of oxidative burst capability, granule release, and the production of chemokines and cytokines [56,58]. The IL-18-mediated priming and enhancement of neutrophil oxidative burst occur through the p38 MAPK pathway [59]. Notable enhancement of oxidative burst by neutrophils leads to greater reactive oxygenated species production, exacerbation of secondary injury, and impaired functional recovery [60]. Additionally, IL-18-mediated granule release containing the destructive protease elastase also occurs through the p38 MAPK pathway [58]. The release of azurophilic granules containing elastase by neutrophils after SCI increases blood-spinal cord barrier disruption and decreases functional recovery following SCI [61]. Furthermore, IL-18 induces neutrophils to release the proinflammatory cytokines IL-1α and TNF-α as well as the chemokine IL-8 [56]. The aforementioned cytokines have been shown to limit regeneration after SCI through increasing neuronal and oligodendrocyte apoptosis as well as driving an inflammatory phenotype shift in macrophages [62,63]. IL-8, or the murine isoform CXCL1, is a potent chemoattractant that further recruits neutrophils to the lesion site and augments demyelination within the CNS [63]. Taken together, IL-18 acts upon neutrophils to drive demyelination and further escalates secondary damages after SCI.

### 3.2. Macrophages

Macrophages represent the next wave of peripheral immune cell infiltration, though their presence in the injury site peaks later, at 7 DPI [14]. While macrophages produce low basal levels of NLRP3 and IL-18, the expression of these proteins depends heavily on their activation state. Macrophages stimulated with IL-4 and IL-13 (anti-inflammatory state) lack Lipopolysaccharide (LPS)-induced NLRP3 expression and have a small increase in LPS-induced IL-18 expression in vitro. However, unstimulated (neutral) or IFN-γ-stimulated (proinflammatory state) macrophages increase their expression of NLRP3 and IL-18 upon LPS stimulation, with proinflammatory macrophages increasing their expression of these proteins to a greater extent than neutral macrophages in vitro [64]. Notably, in the context of SCI, the micropinocytosis of myelin debris by macrophages promotes internal cholesterol crystallization; this process drives ROS production and subsequent NLRP3 inflammasome activation [34]. Parallel to neutrophils, tissue-derived macrophages represent another source of IL-18BP. Stimulation with IFN-γ signals macrophages to produce IL-18BP, making macrophages critical regulators of the IL-18/IFN-γ axis [42].

Macrophages also constitutively express IL-18Rα/β chains and further upregulate their expression when exposed to inflammatory stimuli, such as LPS, TNF-α, IL-1β, IL-12, and IL-18 [65]. In cell culture, anti-inflammatory stimuli upregulated IL-18R in macrophages, as IL-10 enhances IL-18R expression, and when combined with IL-18, the two act in synergy to promote greater expression of IL-18R. When combined with an anti-inflammatory cytokine like IL-10, IL-18 aids in driving macrophages towards an anti-inflammatory state marked by increased expression of CD163 [66]. However, when the extracellular milieu does not contain anti-inflammatory cytokines, IL-18 activates the MEK/ERK1/2 and PI3K/Akt signaling pathways to drive inflammatory cytokine release such as monocyte chemoattractant protein-1 [67].

### 3.3. CD4+ T-Cells

CD4+ T-cells represent another population of peripheral immune cells that invade the injured CNS parenchyma, emerging as the predominant CD45-expressing infiltrating leukocyte 7 DPI [68]. Unlike the aforementioned myeloid cells, CD4+ T-cells function primarily as responders to IL-18 rather than producers. Although these lymphocytes possess the NLRP3 inflammasome machinery and secrete IL-1β, single-cell RNA sequencing from peripheral blood and lung tissue suggests T-cells lack IL-18 mRNA [69,70]. Furthermore, flow cytometry characterization of IL-18BP sources confirms that T-cells do not produce this regulatory protein [42].

As dedicated responders to IL-18, CD4+ T-cells constitutively express IL-18Rα following their differentiation in the thymus. However, both receptor density and the subsequent response to receptor stimulation strictly depend on the activation state and the cytokine milieu driving Th1 (Stat4) or Th2 (Stat6) differentiation. Activated Th1 memory CD4+ T-cells exhibit higher densities of IL-18Rα compared to their naïve counterparts; this high receptor density allows memory CD4+ T-cells to produce IFN-γ in the presence of IL-18 alone. Conversely, naïve CD4+ T cells, which express intermediate amounts of IL-18Rα, require the combined input of T-cell receptor (TCR) stimulation, IL-12, and IL-18 to trigger IFN-γ production. These naïve cells also rely on TCR stimulation, IL-12, and IFN-γ to upregulate IL-18Rα and commit to the Th1 lineage via Stat4 signaling. On the contrary, naïve CD4+ T cells that undergo TCR stimulation in the presence of IL-4 downregulate IL-18Rα, committing to the Th2 lineage via stat6 signaling [71]. Ultimately, while CD4+ T-cells do not generate IL-18, they utilize the IL-18 produced by surrounding macrophages and neutrophils to amplify the IFN-γ response within the SCI. By binding to the IL-18R on CD4+ T-cells, IL-18 activates the p38 MAPK and NF-κβ pathways, leading to the production of IFN-γ [72]. One study describes Th1 CD4+ T-cells’ ability to secrete IFN-γ, which aids in the promotion of axon remodeling and functional recovery after SCI by inducing further secretion of IL-10, an anti-inflammatory cytokine [73]. However, the current data describing IFN-γ’s net effect on SCI prognosis remain non-convergent. The timing of upregulation and varying degrees of IFN-γ secretion yield conflicting anti-inflammatory or proinflammatory effects [74]. Thus, further research is warranted to fully deduce whether IL-18′s IFN-γ amplifying effect in CD4+ T-cells is beneficial for SCI recovery.

### 3.4. Neurons

Unlike the invading immune cells that upregulate signaling components upon activation, resident neurons demonstrate a paradoxical downregulation of IL-18 machinery following trauma (Table 1). Neurons express several key components of the IL-18 signaling pathway, though their expression levels fluctuate significantly across the injury timeline. At 3 DPI in rats, neurons are the most prominent CNS cell type expressing NLRP3 adjacent to the injury epicenter, representing nearly 60% of all NLRP3-positive cells [31]. Furthermore, in vitro studies on healthy embryonic murine cortical neurons confirm the basal expression of both NLRP3 and IL-18 mRNA [75].

Despite basal IL-18 production in healthy states, neuronal IL-18 levels shift drastically as the inflammatory environment progresses in vivo [76]. In a murine intracerebral hemorrhage (ICH) model, roughly 60% of striatal neurons in sham-operated mice produce IL-18; however, this population drops to 20% within the first 12 h after injury and vanishes completely (0%) 24 h and into 3 DPI. Similar trends occur regarding the IL-18R expression in the same injury model. While 45% of striatal neurons in sham-operated mice express the IL-18R, this frequency plummets to 25% within 12 h post-ICH and continues to decline below 20% at 24 h and into 3 DPI [76].

This inhibitory trend following CNS inflammation persists within the spinal cord adjacent nerve injuries as well. At 7 days after L5 spinal nerve ligation (SNL) in rats, neurons within the ipsilateral dorsal horn of the lumbar spinal cord lack both IL-18 and IL-18R, marked by the absence of colocalization between these proteins and the neuronal cell markers NeuN and Map2 [77]. Collectively, these findings indicate that while healthy neurons maintain the capacity for IL-18 signaling, they rapidly suppress these components under inflammatory conditions. Currently, no research in the context of CNS trauma clarifies whether neurons produce IL-18BP; however, given the rapid downregulation of both the ligand and its receptor, neurons likely provide minor, diminishing contributions to the regulation and progression of IL-18-mediated inflammation following CNS trauma.

### 3.5. Microglia

In contrast to the diminishing signaling seen in neurons, microglia and astrocytes emerge as the primary drivers of IL-18 upregulation following trauma. In particular, microglia serve as central hubs for both the production and modulation of this cytokine (Table 1). While in vitro studies of embryonic murine microglia show low basal levels of NLRP3, these cells significantly increased NLRP3 expression when stimulated with LPS or a pro-inflammatory cocktail containing IL-1β, TNF-α and IFN-γ [75]. These results are mirrored in vivo as 3 DPI in the SCI model, where microglia account for 20% of all NLRP3-expressing cells adjacent to the contusion epicenter [31].

Under inflammatory conditions, microglia function as key producers of IL-18. In vitro analysis demonstrates that embryonic murine microglia secrete IL-18 in an NLRP3-dependent manner when triggered by classical inflammasome activators like ATP and nigericin, as NLRP3 or Caspase-1 knockouts nearly abolish secretion of IL-18 [75]. These results are reflected in vivo as microglial IL-18 production progressively increases alongside injury maturation in a variety of CNS injury models. In the murine ICH model, a small proportion of striatal microglia produce IL-18 in sham mice. However, after injury, the fraction of striatal microglia producing IL-18 significantly rises to 50% within the first 12 h after injury and escalates to nearly 100% within 3 DPI [76]. Similar patterns emerge in rat models of experimental migraine and SNL, where immunofluorescence imaging confirms the robust colocalization of IL-18 within IBA-1-positive microglia adjacent to the injured side in the ipsilateral dorsal horn of the spinal cord [77,78].

Studies identify the TLR4/p38-MAPK pathway as the primary driver of microglial IL-18 production. TLR4 agonism via intrathecal LPS administration increases both the number and intensity of IBA-1 and IL-18 labeled cells, whereas TLR inhibition with TAK significantly reduces microglia-derived IL-18 production [77,78]. Additionally, blocking p38 with the inhibitor SB203580 after LPS-induced activation reduces the immunofluorescence intensity of IL-18 and IBA-1 [77]. Colocalization studies confirm that phosphorylated-p38 specifically labels IL-18-producing cells [77,78].

Regarding the regulation of IL-18, no in vivo data currently exists on microglia production of IL-18BP in the context of traumatic CNS injury models. However, in vitro data displays murine spinal microglia constitutively secrete IL-18 BP; notably, this secretion remains stable even after LPS stimulation [45]. While microglia are definitive producers of IL-18, they lack expression of IL-18R. At 7 DPI in an SNL model or 4 days after repeated inflammatory stimulation in an experimental migraine model, microglia in the ipsilateral dorsal horn of the spinal cord show no expression of the IL-18R, as evidenced by the total absence of colocalization between IBA-1 and IL-18R immunofluorescence [77,78].

### 3.6. Astrocytes

While microglia possess the machinery to generate IL-18, astrocytes function primarily as responders to this cytokine in the context of SCI (Table 1). At 3 DPI in a murine SCI model, astrocytes adjacent to the epicenter express only minimal levels of the NLRP3 inflammasome, representing less than 20% of NLRP3-positive cells [31]. In vitro findings corroborate this, as primary astrocytes from neonatal mice brains show little to no NLRP3 expression even after stimulation with a pro-inflammatory cytokine cocktail [75]. Due to their lack of NLRP3 machinery, astrocytes do not produce IL-18. Immunofluorescent labeling in models of SNL and experimental migraine further confirms this, as the astrocytic marker GFAP does not colocalize with IL-18 near the area of induced injury [77,78].

Although they do not produce IL-18, astrocytes likely regulate the availability of free IL-18 through the production of IL-18BP. While no in vivo data regarding astrocytic production of IL-18BP in CNS trauma currently exists, in vitro studies using human astrocytes demonstrate that these cells secrete IL-18BP under both basal and inflammatory conditions. Specifically, stimulation with IL-1β significantly upregulates astrocytic IL-18BP production [44].

Astrocytes serve as the primary responders to IL-18 during CNS inflammation, as they robustly express the IL-18R across various injury models. At 7 DPI in the murine SNL model, GFAP-positive astrocytes adjacent to the lesion in the ipsilateral dorsal horn exhibit clear IL-18R expression [77]. Similarly, in an experimental migraine model, repeated inflammatory injections induce astrocytic activation and a marked increase in IL-18R expression within the medullary dorsal horn. Upon immunofluorescent analysis, IL-18R was found to overlay with GFAP [78]. Mechanistically, IL-18R activation in astrocytes consequentially activates the p65-NF-κβ pathway as IL-18R colocalizes with p-NF-κβ in GFAP-positive cells in the ipsilateral dorsal horn of the lumbar spinal cord 7 DPI in the SNL model [77]. Additionally, the suppression of IL-18 activity inhibits NF-κβ phosphorylation and attenuates astrocytic activation, confirming that NF-κβ activation occurs directly through IL-18 binding to the astrocytic IL-18R [78].

## 4. IL-18 Endogenous Levels After SCI

**Figure 3:**
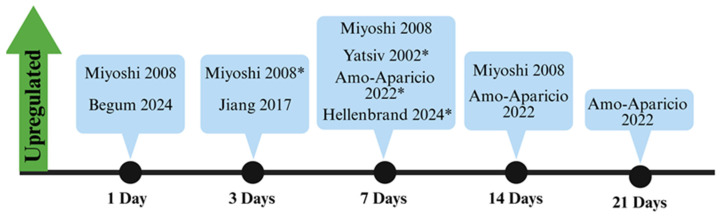
Analysis of temporal timeline of IL-18 levels in the CNS after injury. Majority of studies agree that IL-18 is upregulated following SCI, TBI, and SNL [12,35,77,79,80,81]. Three longitudinal studies confirm IL-18 reaches peak upregulation 7 DPI, two in SCI model and one in TBI model [12,20,35]. Studies were included at timepoints only if significant difference was observed from baseline uninjured control. * indicated peak upregulation of IL-18 from study. Figure created with BioRender 2026.

IL-18′s levels after SCI and other traumatic nervous system injuries are fairly well established (Figure 3). Many authors agree that IL-18 levels increase within the injured spinal cord at later timepoints compared to other inflammatory cytokines, such as IL-6 and IL-1β, which peak within the first 24 h after injury [12]. While Jiang et al. and Begum et al. confirmed IL-18 upregulation at 3- and 7-DPI, respectively, these investigations were limited to single-timepoint analyses [79,80]. In contrast, Hellenbrand et al. and Amo-Aparicio et al. performed longitudinal studies using homogenized spinal cord and revealed consistent temporal timelines with peak IL-18 levels at 7 DPI [12,81]. One paper contradicts these findings, as they show IL-18 to be downregulated after SCI. However, the authors acknowledge that their results contradict the previously published literature and attribute the discrepancy to the small sample size in their study [82].

The temporal timeline of IL-18 established in SCI closely mirrors those observed in other traumatic nervous system injury models, TBI and SNL. Yatsiv et al. mapped the IL-18 timeline in a murine closed-head TBI model using homogenized mouse brains [35]. They reported a timeline parallel to that of SCI, observing a significant peak in IL-18 levels at their final timepoint of 7 DPI [35]. Miyoshi et al. demonstrated a comparable timeline in an SNL model by quantifying the immunofluorescent intensity of IL-18 within the ipsilateral dorsal horn adjacent to the ligated nerve [77]. They found IL-18 to be significantly elevated from 1 to 14 DPI, with 3 DPI marking the peak upregulation and maintaining near-peak values through 7 DPI. Notably, Miyoshi et al. also evaluated IL-18R and phosphorylated NF-κβ immunofluorescent intensities in this same region, discovering that the expression of both factors peaked at 7 DPI [77].

It is important to note that there are different IL-18 quantitative assays on the market. Many assays measure total IL-18, including free IL-18 and IL-18 bound to IL-18BP, while more specialized assays only measure free IL-18 levels [83]. Measurement of total IL-18 does not accurately capture peak free IL-18 levels, as it also measures inactive IL-18 bound to its endogenous inhibitor. Thus, measurement of free IL-18 elucidates the true onset of the cytokine’s effects in disease and injury progression [83]. Future research exploring IL-18 levels and their effect after SCI should implement a free IL-18 quantitative assay while also measuring IL-18BP levels within the injured spinal cord.

## 5. Effects of IL-18 After SCI

Although researchers have quantified IL-18 levels following SCI, basic research elucidating the direct effects of IL-18 on the injury’s pathophysiology remains sparse. Begum et al. demonstrated the effects of IL-18 inhibition in an SCI model, while Miyoshi et al. utilized an SNL model to demonstrate IL-18′s effect on the generation of post-ligation pain [77,80]. To determine the specific impact of IL-18 on SCI, Begum et al. administered a neutralizing anti-IL-18 antibody (IL-18Ab) during the acute phase of injury [80]. Compared to the antibody-treated group, untreated rats exhibited enhanced reactive gliosis, alongside increased infiltration of both microglia/macrophages and neutrophils. Untreated rats also had significantly greater amounts of neuronal loss at the injury epicenter. Furthermore, the researchers observed that IL-18 upregulates Ccl17, an inflammatory marker on microglia/macrophage. Taken together, these findings indicate IL-18 propagates reactive gliosis and drives immune cell infiltration, facilitating neuronal loss after SCI [80].

Beyond the scope of SCI models, Miyoshi et al. demonstrated that IL-18 drives nociceptive pain following SNL [77]. Following ligation, the researchers observed microglia and astrocytes to be in close proximity to each other within the ipsilateral dorsal horn of the spinal cord. These closely associated cells express IL-18 (microglia) and its corresponding receptor, IL-18R (astrocytes). Mechanistically, TLR/MAPK signaling and the subsequent translocation of p38 mediate microglial IL-18 production while IL-18R activation in astrocytes triggers the NF-κβ pathway and p65 translocation. Using selective inhibitors against these pathways, Miyoshi et al. confirmed that the generation of nociceptive pain after SNL relies directly on microglial IL-18 production and its subsequent reception by neighboring astrocytes [77].

Notably, the cellular pathways identified in the SNL model also drive neuropathic pain following SCI and may contribute to astrocytic border formation. Crown et al. observed that the p38-MAPK expression increases and colocalizes with both microglia and astrocytes 35 DPI in murine SCI. Furthermore, selective inhibition of this pathway with SB203580 reduced tactile allodynia [84]. Similarly, adult mouse spinal cords 12 h post-injury revealed p65-NF-κβ translocation within GFAP-expressing astrocytes [85]. Thus, following SCI, microglia and astrocytes actively express p38-MAPK and p65-NF-κβ, respectively.

These distinct signaling pathways likely influence the formation of the astrocytic border, a process structurally characterized by the close proximity of microglia and astrocytes within the epicenter of injury [86]. Umezawa et al. demonstrated that heterozygous p38 knockout mice develop significantly smaller borders than wild-type controls, exhibiting reduced astrocyte accumulation and tighter microglial localization within the lesion [87]. Additionally, Karova et al. demonstrated that implanting spinal progenitor cells into injured rat spinal cords reduced p65-NF-κβ activation and subsequently reduced astrocytic border formation compared to controls [88]. While modulating p38-MAPK and p65-NF-κβ clearly impacts both neuropathic pain and border formation, definitive evidence directly linking IL-18 to the SCI astrocytic border remains elusive. Future research is needed to determine whether microglia and astrocytes within the border actively express IL-18 and IL-18R and employ selective inhibitors to definitively test whether IL-18 signaling between these microglia and astrocytes drives astrocytic border formation.

## 6. Treatments That Modulate IL-18 After SCI

**Figure 4:**
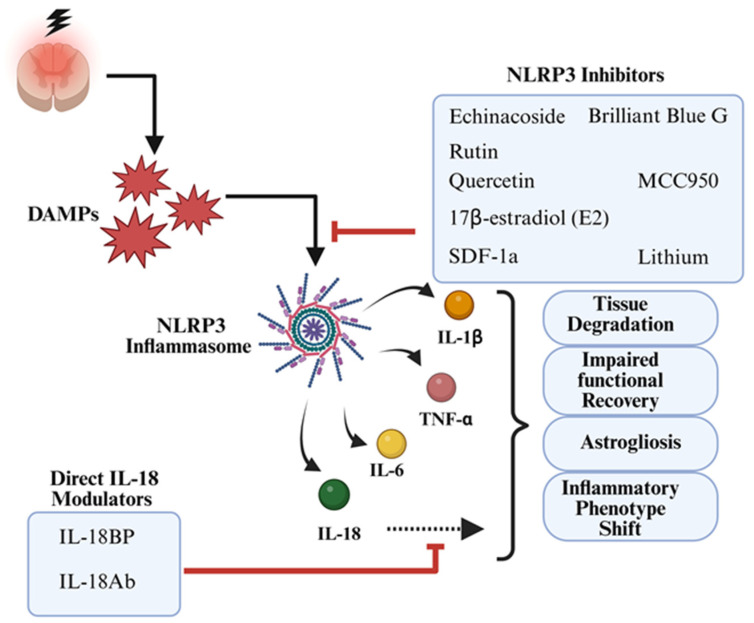
Mechanisms of manipulating IL-18 levels. Following SCI, DAMPs are released, leading to the assembly of the NLRP3 inflammasome and downstream pro-inflammatory cytokine release. Inflammatory cytokine release causes an inflammatory state shift in astrocytes and macrophages, subsequently causing tissue degradation and impaired functional recovery. Echinacoside, Rutin, Quercetin, E2, SDF-1a, Brilliant Blue G, MCC950, and Lithium directly inhibit NLRP3 inflammasome assembly [31,89,90,91,92,93,94,95]. Direct inhibition of IL-18 can be accomplished through IL-18Ab administration or IL-18BP level enhancement [45,80]. Dotted line is IL-18 production. Black arrows refer to process and red lines are inhibition. Figure created with BioRender 2026.

Many different interventions exist to decrease IL-18 levels following SCI, though their mechanism of action occurs at different levels of the processing and reception pathway (Figure 4). Therapeutics that successfully disrupt upstream assembly of the NLRP3 inflammasome also suppress downstream IL-18 production. However, their mechanisms inherently reduce a wider panel of pro-inflammatory cytokines, including TNF-α, IL-1β, and IL-6 [31,89,90,91,92,93,94,95]. Therefore, observed improvements in tissue preservation, cellular phenotype, and functional recovery following inhibition of the NLRP3 inflammasome cannot be exclusively attributed to IL-18 suppression. However, therapeutics that directly inhibit IL-18, or modulate endogenous inhibitors of IL-18, can attribute observed improvements directly to IL-18 suppression.

### 6.1. NLRP3 Inhibition

Modulation of the NLRP3 inflammasome has been shown to influence proinflammatory cytokine release, including IL-18, following SCI. Researchers have deployed a variety of treatments to manipulate this complex, including medicinal natural products, endogenous sex steroids, textile dyes, chemical elements, and synthetic selective inhibitors.

**Figure 5:**
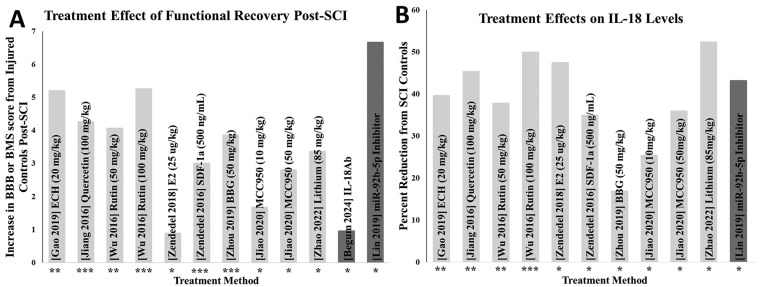
Quantitative effects of treatments that reduce IL-18 after SCI. (**A**) Effect of treatments on functional recovery following SCI. Functional recovery scores were compared between injured controls and treatment groups at the final timepoint of each study; ECH: 35 DPI [89]; quercetin: 14 DPI [90]; rutin: 14 DPI [91]; E2: 3 DPI [92]; SDF-1a: 7 DPI [31]; BBG: 21 DPI [93]; MCC950: 28 DPI [94]; Lithium: 28 DPI [95]; IL-18Ab: 42 DPI [80]; miR-92b-5p inhibitor: 14 DPI [45]. (**B**) Effects of treatments on IL-18 levels following SCI. IL-18 levels were compared between untreated injured controls and treatment group at timepoint of comparison specific to each study; ECH: 3 DPI [89]; quercetin: 3 DPI [90]; rutin: 3 DPI [91]; E2: 3 DPI [92]; SDF-1a: 3 DPI [31]; BBG: 3 DPI [93]; MCC950: 7 DPI [94]; lithium: 7 DPI [95]; miR-92b-5p inhibitor: 3 DPI [45]. Light gray bars indicate NLRP3 manipulation; dark gray bars indicate direct IL-18 inhibition. * (*p* < 0.05); ** (*p* < 0.01); *** (*p* < 0.001) compared to untreated injured controls.

Among the natural products, echinacoside (ECH) demonstrates the ability to suppress NLRP3 priming and activation. Gao et al. evaluated this herbal compound, revealing significant in vitro efficacy that translated to an in vivo murine SCI model [89]. In vitro, ECH dose-dependently inhibited ROS generation and NF-κβ phosphorylation in BV-2 cells (microglia cell line) subjected to inflammatory stimuli. The anti-inflammatory profile of ECH processes also successfully carried over to in vivo SCI applications, as daily intraperitoneal (IP) injections of ECH following SCI significantly downregulated the expression of inflammasome components NEK7, NLRP3, ASC, and Caspase-1 compared to untreated controls at 3 DPI. Consequently, ECH administration significantly attenuated IL-18 production at this same timepoint. By suppressing this inflammatory cascade, ECH preserves tissue architecture, yielding significantly more intact neurons at 7 DPI. This combined reduction in inflammation-inducing machinery and enhanced neuronal survival improves functional recovery; ECH-treated animals achieved significantly higher Basso, Beattie, and Bresnahan (BBB) locomotor scores starting at 7 and through 35 DPI (Figure 5A). Taken together, ECH is a viable therapeutic for inhibiting NLRP3 inflammasome assembly and restoring functional recovery after SCI [89].

Building upon the therapeutic potential of natural products, plant-derived flavonoids, quercetin (Jiang et al.) and rutin (Wu et al.), offer parallel neuroprotective effects in rat compression SCI models [90,91]. IP administration of either compound immediately following SCI and sustained treatment over the acute phase of injury disrupts the upstream triggers of IL-18 maturation. Both quercetin and rutin treatment significantly attenuate oxidative stress within the injured spinal cord, evidenced by marked reductions in ROS production at 3 DPI compared to untreated injured controls. By neutralizing oxidative stress, these flavonoids effectively suppress the assembly of the NLRP3 inflammasome, as treated tissues exhibit significantly diminished levels of NLRP3, ASC, and active caspase-1 as early as 24 h post-injury. Consequently, this blockade inhibits the downstream production of IL-18 and preserves structural tissue architecture (Figure 5B). Histological evaluations at 3 DPI reveal that both flavonoids significantly lower pathological scores by mitigating edema, neutrophil infiltration, hemorrhage, and congestion within the injured spinal cord. Ultimately, the morphological sparing rescues motor function, enabling both quercetin- and rutin-treated animals to have significantly higher BBB locomotor scores from 3 to 14 DPI (Figure 5A). Collectively, these studies illustrate that neutralizing upstream oxidative stress with flavonoids can mitigate NLRP3 activation, restrict IL-18 secretion, promote tissue sparing, and recovery [90,91].

Transitioning from exogenous plant-derived flavonoids to endogenous signaling molecules, sex steroids offer a promising avenue for modulating NLRP3 activation and downstream IL-18 production. Zendedel et al. explored this by administering 17β-estradiol (E2) to male Wistar rats subjected to a contusion SCI [92]. Repeated subcutaneous injections of E2, initiated immediately post-injury and administered every 12 h, effectively inhibited the activation of the inflammasome cascade over the course of 3 DPI. E2 treatment significantly downregulated the spinal mRNA expression of the inflammasome components NLRP3 and ASC. Consequently, this upstream suppression significantly reduced IL-18 mRNA and protein levels at 3 DPI (Figure 5B). By suppressing the local inflammasome activation, E2 preserved the cellular landscape at the injury epicenter 3 DPI; treated tissues exhibited a significant reduction in neuroinflammatory microglia alongside a significant increase in myelin-producing oligodendrocytes. The shift in cell populations present in the epicenter underscores how manipulating inflammasome assembly after SCI can restrict inflammation caused by inflammatory immune cells and preserve myelin-producing cells following injury. Furthermore, the reduction in inflammatory machinery and the preservation of myelinating cells accelerated early functional recovery, with E2-treated animals having significantly higher BBB scores at 3 DPI (Figure 5A). Collectively, these findings position E2 as a promising therapeutic capable of dismantling inflammatory cytokine-producing machinery, mitigating local neuroinflammation, and promoting early functional recovery after SCI [92].

Alongside endocrine signaling, endogenous cytokines such as stromal cell-derived factor-1 alpha (SDF-1a), a chemoattractant known to rescue neural apoptosis, demonstrate regulatory control over the NLRP3 inflammasome. Zendedel et al. elucidated the cytokine’s neuroprotective capacity in a rat compression SCI model [31]. Sustained administration of SDF-1a inhibited acute inflammasome assembly. By 3 DPI, SDF-1a treatment significantly downregulated the mRNA and protein expression of NLRP3 and ASC within the spinal cord. Treated rats also displayed significantly lower amounts of active caspase-1 compared to untreated controls at the same timepoint as the NLRP3 components. Together, these results indicate SDF-1a inhibits NLRP3 inflammasome assembly, inhibiting the catalytic conversion of procaspase- into its catalytically active form. Consequently, this blockade restricted the downstream maturation of IL-18, significantly reducing both its transcription and protein accumulation within the spinal cord at 3 DPI (Figure 5B). Notably, this acute inflammatory suppression orchestrated beneficial shifts in the immune landscape during the chronic phase of SCI. At 28 DPI, SDF-1a-treated tissues exhibited elevated numbers of IBA1+ microglia; however, rather than adopting the rounded, amoeboid shape characteristic of an inflammatory state, these microglia maintained a highly ramified, neuroprotective morphology. This phenotypic shift was quantitatively supported by significantly elevated ratios of protective (Arginase 1-expressing) to inflammatory (iNOS-expressing) mRNA profiles in treated rats at 28 DPI. Collectively, inhibiting acute IL-18 levels while maintaining a chronic protective microglial environment translated into accelerated functional recovery, as SDF-1a administration yielded significantly higher BBB scores from 3 through 7 DPI (Figure 5A). Taken together, SDF-1a is a promising therapeutic to modulate IL-18 levels after SCI as it inhibits upstream NLRP3 inflammasome assembly, decreases IL-18 levels, and improves functional recovery in the acute phases of injury while shifting microglia towards a protect state in the chronic phase of injury [31].

Beyond endogenous factors, synthetic compounds such as the textile dye Brilliant Blue G (BBG) offer another distinct pharmacological mechanism for NLRP3 inhibition and IL-18 level modulation following SCI. BBG functions as a selective antagonist of the P2X7 receptor, an ATP-gated ion channel. By blocking this receptor, BBG prevents the injury-induced intracellular cascades that normally trigger NLRP3 inflammasome activation [93]. Zhou et al. demonstrated the translational potential of BBG in an adult male rat compression SCI model [93]. IP administration of BBG 20 min post-injury and maintaining administration every 12 h robustly suppressed the inflammasome cascade over the acute 3 DPI period. At 3 DPI, BBG treatment significantly decreased the expression of inflammasome-associated proteins within the spinal cord, including P2X7, NLRP3, ASC, cleaved XIAP, caspase-11, and caspase-1. Correspondingly, this upstream blockade significantly decreased IL-18 production at the same timepoint (Figure 5B). The suppression of inflammasome proteins and IL-18 translated directly to tissue preservation. Histological evaluations revealed that BBG significantly mitigated secondary tissue damage, marked by less spinal cord congestion, edema, neutrophil infiltration, and structural disruption compared to untreated injured controls. Tissue sparing enabled higher motor recovery, with BBG-treated rats exhibiting significantly higher BBB locomotor scores from 7 through 21 DPI (Figure 5A). In conclusion, BBG is a promising therapeutic capable of regulating IL-18 secretion via P2X7/NLRP3 inhibition, restricting histological damage and driving functional repair [93].

Another synthetic compound found to manipulate IL-18 levels following SCI is MCC950, a highly selective NLRP3 inhibitor. Jiao et al. evaluated this targeted approach in a murine SCI model, demonstrating its capacity to improve functional recovery [94]. In vitro, MCC950 effectively blocks the assembly of the inflammasome marked by inhibition of NLRP3-ASC and NLRP3-caspase-1 complexes. In vivo, IP administration of MCC950 at 1 and 3 h post-injury significantly reduced systemic serum IL-18 levels at 7 DPI. The attenuation of acute, systemic inflammation translated into marked motor improvements. MCC950-treated mice displayed significantly higher Basso Mouse Scale (BMS) locomotor scores and enhanced forelimb grip strength from 7 through 28 DPI compared to injured controls (Figure 5A). Collectively, MCC950 decreases the inflammatory response in the acute phase of SCI and improves functional recovery in murine SCI by inhibiting NLRP3 inflammasome assembly, making it a suitable treatment for alleviating downstream neuroinflammation caused by IL-18 [94].

Transitioning from complex pharmacological compounds, the simple chemical element lithium provides another potent mechanism for regulating IL-18 production via the suppression of upstream oxidative stress. Zhao et al. demonstrated its therapeutic potential in a murine T10 contusion model with the administration of a single IP injection of lithium chloride shortly after the initial injury [95]. By 7 DPI, this single intervention profoundly neutralized oxidative stress within the spinal cord and effectively downregulated the regional expression of NLRP3, ASC, and active caspase-1. By suppressing these core components, lithium inhibited local pyroptotic pathways and significantly decreased the accumulation of IL-18 at 7 DPI (Figure 5B). Furthermore, lithium treated rat had significantly less neuronal damage in the epicenter of the injury at the same timepoint. The significant structural sparing translated into accelerated motor recovery. Lithium-treated animals achieved significantly higher BBB locomotor scores starting at 5 DPI and maintained significantly elevated scores through 28 DPI (Figure 5A). Collectively, lithium’s antioxidant properties allow it to regulate IL-18 levels following injury by suppressing NLRP3 inflammasome assembly. In turn, suppression of NLRP3 assembly mitigates pyroptotic neuronal death and drives functional repair after SCI [95].

### 6.2. Direct IL-18 Neutralization

While the aforementioned therapeutics successfully disrupt upstream NLRP3 inflammasome assembly to suppress IL-18, their broad mechanisms inherently reduce a wider panel of pro-inflammatory cytokines. The treatments listed below take a more direct approach by binding free IL-18 or inhibiting its complementary receptor, IL-18R.

Begum et al. investigated the therapeutic efficacy of IL-18Ab in an adult mouse T9 dorsal hemi-section model [80]. To directly neutralize circulating IL-18, researchers administered IP injections of IL-18Ab at 1, 3, and 7 DPI. The direct inhibition altered the local neuroimmune response. By 3 DPI, IL-18Ab treatment significantly attenuated reactive gliosis and restricted microglial accumulation at the injury epicenter, evidenced by marked decreases in GFAP immunofluorescent intensity and IBA1-positive cell counts. Furthermore, neutralizing free IL-18 shifted immune cell polarization. IL-18Ab effectively suppressed the early pro-inflammatory response by downregulating the mRNA transcription of IL-1β and Ccl17. Concurrently, IL-18Ab enhanced the anti-inflammatory response through upregulation of Arginase-1 mRNA, an anti-inflammatory marker. Restriction of the acute inflammatory cascade conferred significant structural neuroprotection. At 7 DPI, IL-18Ab administration prevented neuronal apoptosis rostral to the lesion, significantly reducing the population of cells co-expressing NeuN and cleaved caspase-3. This immune modulation and anatomical sparing yielded modest improvements in motor function as IL-18Ab-treated mice achieved significantly higher BMS scores at 14 and 42 DPI (Figure 5A). Taken together, direct inhibition with IL-18Ab mitigates reactive gliosis, drives protective microglial polarization, and rescues neurons, facilitating functional recovery after SCI [80].

Parallel to directly neutralizing the cytokine, upregulating its endogenous decoy receptor, IL-18BP, provides another highly targeted avenue for suppressing free IL-18 following SCI. Lin et al. identified MiR-92b-5p as a regulator of IL-18 upregulation [45]. They demonstrated that MiR-92b-5p is significantly upregulated following injury, a shift which correlates with the increase in IL-18 and the concurrent suppression of IL-18BP mRNA production. To reverse this imbalance, researchers administered an intrathecal injection of a MiR-92b-5p inhibitor following a contusion SCI in mice. By 3 DPI, the inhibitor significantly suppressed IL-18 production while upregulating endogenous IL-18BP production. By enhancing the natural sequestration of free IL-18, the MiR-92b-5p inhibitor enhanced motor recovery, as treated mice had significantly higher BMS scores at 3 and through 14 DPI (Figure 5A). Ultimately, inhibition of MiR-92b-5p effectively mitigates IL-18-driven inflammation by increasing IL-18BP, presenting a potent genetic strategy for functional repair [45].

Direct inhibition of IL-18 has also been shown to be beneficial outside the scope of SCI and in other neuroinflammation-inducing injury models. Gong et al. demonstrated the broad efficacy of IL-18 neutralization in a murine experimental migraine model [78]. To induce localized neuroinflammation, researchers subjected adult male rats to daily subdural injections of an inflammatory cocktail containing histamine, serotonin, bradykinin, and prostaglandin E2 over four consecutive days. IP administration of IL-18Ab 1.5 h prior to each subdural injection provided robust effects. IL-18Ab treatment significantly reduced IL-18 levels and the expression of astrogliosis markers, GFAP and p-p65, within the medullary dorsal horn. By suppressing localized astrogliosis, the IL-18Ab mitigated migraine-induced pain behaviors; treated rats exhibited significantly less facial rubbing and maintained a much higher withdrawal threshold during von Frey filament testing. These findings collectively illustrate that direct inhibition of free IL-18 not only mitigates astrogliosis but also alleviates severe nociceptive hypersensitivity driven by CNS neuroinflammation [78].

Further corroborating these pain-mitigating effects, Miyoshi et al. explored direct IL-18 and IL-18R inhibition in an adult male rat L5 SNL model [77]. Notably, IL-18Ab was not the only method used to modulate IL-18-induced effects; the authors also used an anti-IL-18R antibody (IL-18RAb) and IL-18BP. Delivering these treatments intrathecally via subdural pumps, researchers evaluated both prophylactic and delayed administration. For the prophylactic approach, continuous delivery of either IL-18Ab, IL-18RAb, or IL-18BP was initiated 12 h prior to SNL and maintained through 7 DPI. By 7 DPI, pre-treatment with IL-18Ab and IL-18RAb significantly decreased astrocyte activation and proliferation, evidenced by markedly reduced immunofluorescent intensity of GFAP and p-NF-κβ in the ipsilateral dorsal horn. Consequentially, all three prophylactic treatments significantly reduce tactile allodynia in the ipsilateral paw. The authors also demonstrated the powerful efficacy of a delayed intervention. Initiating continuous intrathecal IL-18BP delivery at 7 DPI, after pathological pain states were already established, significantly reversed SNL-induced mechanical hypersensitivity from 10 to 14 DPI. These findings confirm that inhibition of IL-18/IL-18R activation in glial cells can inhibit astrogliosis and reduce nociceptive pain in murine SNL [77].

Although IL-18 modulators reduce secondary damage and restore a significant amount of hindlimb function following SCI in rodents, residual functional deficits persist after injury. These findings underscore the need for combinatorial therapeutic strategies that not only attenuate secondary damage but also promote neural regeneration following SCI.

## 7. Conclusions

The secondary damage following SCI exacerbates the inflammatory process. While acute inflammatory cytokines reach peak within 24 h after injury, we conclude IL-18 to be an outlier, peaking at a later time point, 7 DPI. IL-18 production in SCI is initiated by the priming and activation of the NLRP3 inflammasome. Sequentially, IL-18 is primarily produced by microglia via the p38-MAPK pathway and is received by astrocytes, driving pain, reactive gliosis, and neuronal apoptosis. All treatments modulating either the NLRP3 inflammasome or direct IL-18 inhibition improved functional recovery scores, indicating manipulation of IL-18 with therapeutics is a viable strategy to increase tissue preservation and functional recovery following SCI.

## Figures and Tables

**Table 1:** Expression of IL-18 signaling components during CNS inflammation.

Cell Type	NLRP3	IL-18	IL-18R	IL-18BP
Neurons	+ ^1^	− ^1^	− ^1^	?
Microglia	+ ^1,2^	+ ^1^	− ^1^	+ ^2^
Astrocytes	− ^1,2^	− ^1^	+ ^1^	+ ^2^

^1^ Supported by in vivo data. ^2^ Supported by in vitro data.

## Data Availability

No new data were created or analyzed in this study.

## Abbreviations

The following abbreviations are used in this manuscript:

ASC	Apoptosis-associated Speck-like protein containing a Caspase recruitment domain
BBB	Basso, Beattie, and Bresnahan
BBG	Brilliant Blue G
BMS	Basso Mouse Scale
CNS	Central Nervous System
DAMPs	Damage-Associated Molecular Patterns
DPI	Days Post-Injury
E2	17β-estradiol
ECH	Echinacoside
ECM	Extracellular Matrix
ICH	Intracerebral Hemorrhage
IFN-γ	Interferon-γ
IL	Interleukin
IL-18Ab	Anti-IL-18 Antibody
IL-18BP	IL-18 binding protein
IL-18R	IL-18 receptor
IL-18RAb	Anti-IL-18R Antibody
IRAK1	IL-1R-associated kinase 1
IRAK4	IL-1R-associated kinase 4
LPS	Lipopolysaccharide
MAPK	Mitogen-Activated Protein Kinase
MyD88	Myeloid Differentiation primary response protein 88
NEK7	NIMA-related kinase 7
NF-κβ	Nuclear Factor-κβ
NLRP1	NOD-Like Receptor family Pyrin domain containing 1
NLRP3	NOD-Like Receptor family Pyrin domain containing 3
PI3K	Phosphoinositide 3-kinase
ROS	Reactive Oxygen Species
SCI	Spinal Cord Injury
SDF-1a	Stromal cell-derived factor-1 alpha
SNL	Spinal Nerve Ligation
TBI	Traumatic Brain Injury
TCR	T-cell Receptor
Th	T helper cell
TIR	Toll/IL-1 receptor
TLR	Toll-Like Receptor
TNF-α	Tumor Necrosis Factor-α
TRAF6	Tumor Necrosis Factor Receptor-Associated Factor 6
TRAM	TRIF-related adaptor molecule
XIAP	X-linked inhibitor of apoptosis protein
